# Inverse Stage Migration in Radical Prostatectomy—A Sustaining Phenomenon

**DOI:** 10.3389/fsurg.2021.612813

**Published:** 2021-03-01

**Authors:** Benedikt Hoeh, Felix Preisser, Philipp Mandel, Mike Wenzel, Clara Humke, Maria-Noemi Welte, Matthias Müller, Jens Köllermann, Peter Wild, Luis A. Kluth, Frederik C. Roos, Felix K. H. Chun, Andreas Becker

**Affiliations:** ^1^Department of Urology, University Hospital Frankfurt, Frankfurt, Germany; ^2^Cancer Prognostics and Health Outcomes Unit, Division of Urology, University of Montréal Health Center, Montréal, QC, Canada; ^3^Dr. Senckenberg Institute of Pathology, University Hospital Frankfurt, Frankfurt, Germany

**Keywords:** prostate cancer, radical prostatecomy, D'Amico classification, inverse stage migration, Gleason score

## Abstract

**Objective:** To investigate temporal trends in prostate cancer (PCa) radical prostatectomy (RP) candidates.

**Materials and Methods:** Patients who underwent RP for PCa between January 2014 and December 2019 were identified form our institutional database. Trend analysis and logistic regression models assessed RP trends after stratification of PCa patients according to D'Amico classification and Gleason score. Patients with neoadjuvant androgen deprivation or radiotherapy prior to RP were excluded from the analysis.

**Results:** Overall, 528 PCa patients that underwent RP were identified. Temporal trend analysis revealed a significant decrease in low-risk PCa patients from 17 to 9% (EAPC: −14.6%, *p* < 0.05) and GS6 PCa patients from 30 to 14% (EAPC: −17.6%, *p* < 0.01). This remained significant even after multivariable adjustment [low-risk PCa: (OR): 0.85, *p* < 0.05 and GS6 PCa: (OR): 0.79, *p* < 0.001]. Furthermore, a trend toward a higher proportion of intermediate-risk PCa undergoing RP was recorded.

**Conclusion:** Our results confirm that inverse stage migration represents an ongoing phenomenon in a contemporary RP cohort in a European tertiary care PCa center. Our results demonstrate a significant decrease in the proportion of low-risk and GS6 PCa undergoing RP and a trend toward a higher proportion of intermediate-risk PCa patients undergoing RP. This indicates a more precise patient selection when it comes to selecting suitable candidates for definite surgical treatment with RP.

## Introduction

After the introduction of prostate-specific antigen (PSA) and its use for screening and early detection of prostate cancer (PCa), changes of disease characteristics in PCa patients have been recorded. Several studies reported an increased prevalence of localized PCa patients with a migration toward earlier-stage cancer being diagnosed in younger patients with lower preoperative PSA values as well as an increase in the proportion of patients with organ confined disease at final pathology after radical prostatectomy (RP) (1–4). This phenomenon is widely known as PCa stage migration. Due to the relatively low cancer-specific mortality of low-risk PCa and the potential side effects of RP, such as urinary incontinence and erectile dysfunction, concerns of overtreatment have been raised (5, 6). In consequence, the concept of active surveillance and eventually deferred treatment has gained increased popularity and nowadays represents a standard treatment recommendation in current guidelines (7, 8).

However, with the growing popularity of multimodal treatment strategies for advanced and high-risk PCa, a study published in 2008 firstly demonstrated an inverse trend toward locally advanced tumors in a multinational cohort of patients treated with RP (9). This trend—known as inverse stage migration—was accompanied by an increased rate of patients harboring high-grade PCa at clinical and histopathological characteristics and was confirmed in various studies (10–12).

With uprising insights in multidisciplinary therapies, ongoing establishment of systemic agents, and new focal therapeutic approaches, patient selection is—more than ever—considered to be the key to provide the best available therapy while treating PCa patients.

Taking this into consideration, we assessed the trend of patients, which underwent RP at our institution. We propose that a decrease in the amount of RP in the low-risk constellation can be seen as an indiciator for a proper patient selection.

## Materials and Methods

### Study Population

PCa patients who underwent RP between January 2014 and December 2019 (*n* = 550) were retrospectively identified from our prospectively collected institutional database (Department of Urology, University Hospital Frankfurt, Frankfurt am Main, Germany). All patients had given written consent, and the study was approved by the local institutional review boards of the University Cancer Center Frankfurt and the Ethical Committee at the University Hospital Frankfurt.

Patients treated with neoadjuvant androgen deprivation (*n* = 15) or radiotherapy prior to RP (*n* = 2) were excluded from our study. Subsequently, we excluded patients with missing clinical characteristics to perform D'Amico risk stratification (*n* = 5), resulting in 528 patients, which represent the focus of the current analysis.

### Study Design

All patients underwent either an open retropubic or robotic-assisted laparoscopic transperitoneal RP. Patients were stratified according to the D'Amico risk stratification (13) and Gleason score (GS) (14). Specifically, low-risk PCa was defined as PSA < 10 ng/ml, GS < 7, and clinical stage ≤ T2a; intermediate risk PCa was defined as PSA 10–20 ng/ml and/or GS 7 and/or clinical stage ≤ T2b. Conversely, high-risk PCa was defined as PSA > 20 ng/ml and/or GS > 7 and/or clinical stage ≥T2c (7).

### Statistical Analyses

Descriptive statistics included frequencies and proportions for categorical variables. Medians and interquartile ranges (IQR) were reported for continuously coded variables.

Annual trends of performed surgeries were plotted, after stratification according to the D'Amico risk classification for the low-, intermediate-, and high-risk cohort and for the Gleason score (GS6, GS7, and GS8–10), adjustment was performed for the annual surgical volume. The estimated annual percentage change (EPAC) was calculated for every cohort between 2014 and 2019.

Subsequently, two sets of multivariable logistic regression models were fitted to test the relationship between more contemporary year of surgery and the odds to undergo RP. Specifically, the first set tested the relationship between year of surgery and the D'Amico risk stratification (low, intermediate, and high risk). Here, all multivariable models were adjusted for year of surgery, age at surgery, body mass index (BMI), and Charlson comorbidity index (CCI). Conversely, the second set tested the relationship between year of surgery and the Gleason score (GS6, GS7, and GS8–10). Here, all multivariable models were adjusted for year of surgery, age at surgery, BMI, CCI, and PSA.

All models were repeated with age treated as a categorical coded variable ( ≤ 60 vs. >60– ≤ 70 vs. >70 years).

R software environment for statistical computing and graphics (R Foundation for Statistical Computing, Vienna, Austria, version 3.4.0 for MAC OS X) was used for all statistical analyses. All tests were two sided with a level of significance set at *p* < 0.05.

## Results

### Descriptive Characteristics

Overall, 528 patients underwent RP between 2014 and 2019 with histologically confirmed PCa. Patient and preoperative tumor characteristics are summarized in Table 1. The overall median age at the time of RP was 67 years (IQR: 62–71 years), median preoperative PSA was 8.2 ng/ml (IQR: 6.0–12.1 ng/ml), and median BMI was 26.1 kg/m^2^ (IQR: 24.1–29.1). Overall, 305 (57.8%) patients harbored a CCI of 0, 125 (23.7%) patients harbored a CCI of 1, and 67 (12.7%) patients harbored a CCI of 2, leaving 31 (5.8%) patients with a CCI >2.

**Table 1:** Preoperative characteristics of patients undergoing radical prostatectomy between 2014 and 2019.

	**All patients *n* = 528**
Age (yrs), median (IQR)	67 (62–71)
Body Mass Index (kg/m^2^), median (IQR)	26.1 (24.1–29.1)
Preoperative PSA (ng/ml), median (IQR)	8.2 (6–12.1)
**Charlson Comorbidity Index (%)**	
CCI 0	305 (57.8)
CCI 1	125 (23.7)
CCI 2	67 (12.7)
CCI >2	31 (5.8)
**Clinical stage (%)**	
T1	260 (49.2)
T2	252 (47.7)
T3	16 (3.1)
**D'Amico risk classification (%)**	
Low-risk	60 (11.4)
Intermediate-risk	310 (58.7)
High-risk	158 (29.9)
**Gleason score at biopsy (%)**	
GS6	103 (19.5)
GS7	318 (60.2)
GS 8–10	107 (20.3)

After stratification according to the D'Amico classification, 60 (11.4%) patients were defined as low-risk, 310 (58.7%) as intermediate-risk, and 158 (29.9%) as high-risk PCa patients. After stratification of patients according to the initial histopathology, 103 (19.5%) patients harbored a biopsy of GS6, 318 (60.0%) patients harbored a biopsy of GS7, and 107 (20.0%) patients harbored a biopsy of GS8–10.

### Temporal Trends Stratified According to D'Amico Classification

**Figure 1:**
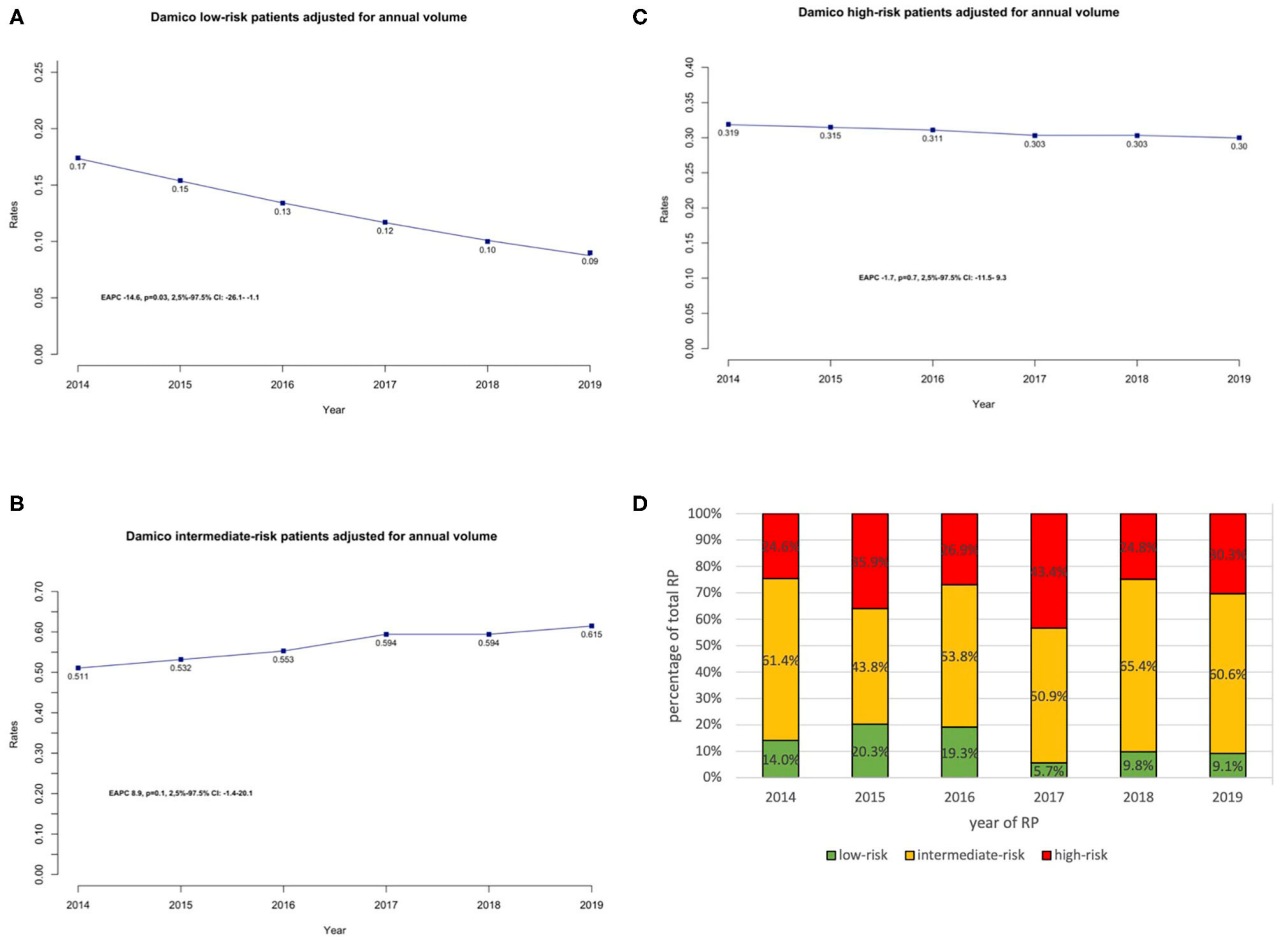
**(A–C)** Trends of performed RP stratified regarding D'Amico low-risk constellation (**A**: low-risk, **B**: intermediate-risk, **C**: high-risk D'Amico classification), adjusted for the annual surgical volume. EAPC was calculated for each cohort separately. **(D)** Proportion of 528 patients stratified according to D'Amico risk groups (low, intermediate, and high risk) shown as a percentage of the total number of cases per year.

From 2014 to 2019, annual rates of patients that underwent RP with D'Amico low-risk characteristics decreased significantly from 17.0 to 9.0% [EAPC: −14.6%, 95% confidence interval (95% CI): −26.1 to −1.1%, *p* < 0.05] (Figure 1A). In multivariable logistic models (Table 2), year of surgery represented an independent predictor to undergo surgery with D'Amico low-risk [odds ratio (OR): 0.85, 95% CI: 0.73–0.99, *p* < 0.05]. Moreover, older patients were less likely to undergo RP with D'Amico low risk (OR: 0.93, 95% CI: 0.89–0.97, *p* < 0.001). After stratifying low-risk patients' age into <60 years vs. 60– ≤ 70 and >70 years, patients who are 60– ≤ 70 years (OR: 0.38, 95% CI: 0.20–0.71, *p* < 0.01) and >70 years (OR: 0.27, 95% CI: 0.11–0.58, *p* < 0.01) were less likely to undergo RP with low-risk PCa compared to patients aged <60 years.

**Table 2:** Multivariable logistic regression models predicting to undergo RP, stratified according to D'Amico risk classification with age continuously coded.

	**Low-risk**			**Intermediate-risk**			**High-risk**		
	**Odds ratio**	**CI: 2.5–97.5%**	***p*-value**	**Odds ratio**	**CI: 2.5–97.5%**	***p*-value**	**Odds ratio**	**CI: 2.5–97.5%**	***p*-value**
Year of surgery	0.85	0.73–0.99	<0.05	1.11	1.01–1.22	<0.05	0.97	0.87–1.09	0.6
Age^*^	0.93	0.89–0.97	<0.001	0.99	0.97–1.02	0.5	1.05	1.02–1.08	<0.001
BMI	0.97	0.90–1.04	0.4	1.00	0.96–1.05	0.9	1.01	0.97–1.06	0.6
CCI 0 (reference)	1			1			1		
CCI 1	1.16	0.57–2.25	0.67	0.89	0.58–1.37	0.6	1.16	0.73–1.82	0.5
CCI 2	0.98	0.38–2.25	0.96	1.20	0.69–2.11	0.5	0.87	0.45–1.57	0.7
CCI >2	0.30	0.02–1.53	0.25	1.19	0.56–2.64	0.7	1.18	0.52–2.57	0.7

* *Continously coded*.

*CI, confidence interval; BMI, Body mass Index; CCI, Charlson Comorbidity Index*.

In the same period of time, the proportion of intermediate-risk patients undergoing RP increased from 51.5 to 61.5%. However, this trend was not statistically significant (EAPC: 8.9%, 95% CI: −1.4 to −20.1%, *p* = 0.1) (Figure 1B). After adjusting for age at surgery, BMI, and CCI, year of surgery did represent an independent predictor to undergo surgery with D'Amico intermediate risk (OR: 1.11, 95% CI: 1.01–1.22, *p* < 0.05), in multivariable logistic models (Table 2). Here, age was no independent predictor to undergo surgery.

Annual rates of patients that underwent RP with D'Amico high-risk characteristics increased from 24.6 to 30.3%. However, this trend was not statistically significant (EAPC: −1.7%, 95% CI: −11.5 to 9.3%, *p* = 0.7) (Figure 1C). Moreover, in multivariable logistic models (Table 2), year of surgery did not represent an independent predictor to undergo surgery with D'Amico high risk (OR: 0.97, 95% CI: 0.87–1.09, *p* = 0.6). However, older age was a significant predictor to undergo RP with high-risk PCA (OR: 1.05, 95% CI: 1.02–1.08, *p* < 0.001). In particular, patients with an age of above 70 years showed a significant tendency to undergo RP with high-risk PCa (OR: 2.21, 95% CI: 1.27–3.92, *p* < 0.01), compared to younger patients.

Figure 1D displays the distribution of D'Amico risk groups as a percentage of the total of number of RP performed each year.

BMI and CCI did not represent significant independent predictors for undergoing RP in each D'Amico risk stratification subgroups.

### Temporal Trends Stratified According to Gleason Score

**Figure 2:**
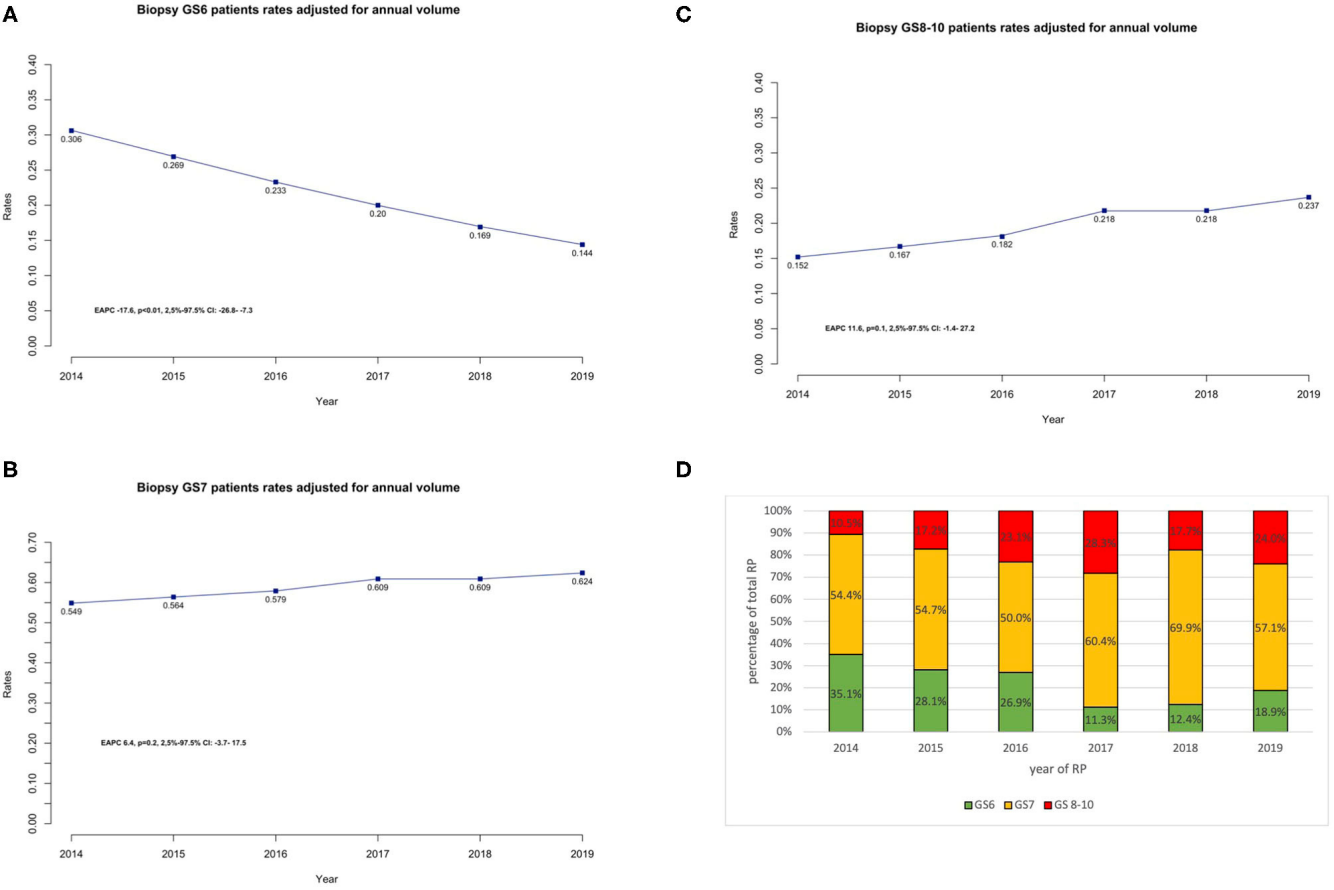
**(A–C)** Trends of performed RP stratified to Gleason score at time of biopsy (**A**: GS6, **B**: GS7, **C**: GS8–10), adjusted for the annual surgical volume. EAPC was calculated for each cohort separately. **(D)** Proportion of 528 patients stratified according to Gleason score at time of biopsy (GS6, GS7, GS8–10) shown as a percentage of the total number of cases per year.

Annual rates of patients that underwent RP harboring GS6 at initial biopsy statistically significantly decreased (EAPC: −17.6%, 95% CI: −26.8 to −7.3%, *p* < 0.01) (Figure 2A). Moreover, in multivariable logistic models (Table 3), more recent year of surgery represented an independent predictor to undergo surgery with a GS6 histology (OR: 0.79, 95% CI: 0.69–0.89, *p* < 0.001). Likewise, older age represented an independent protective predictor to undergo surgery with a GS6 histology (OR: 0.96, 95% CI: 0.93–0.99, *p* < 0.05). After stratifying GS6's patients age into <60 vs. 60– ≤ 70 and >70 years, patients 60– ≤ 70 years (OR: 0.57, 95% CI: 0.33–1.00 *p* < 0.05) and >70 years (OR: 0.42, 95% CI: 0.21–0.82, *p* < 0.05) were less likely to undergo RP with GS6 compared to patients <60 years.

**Table 3:** Multivariable logistic regression models predicting to undergo RP, stratified according to Gleason score with age continuously coded.

	**GS6**			**GS7**			**GS 8–10**		
	**Odds ratio**	**CI: 2.5–97.5%**	***p*-value**	**Odds ratio**	**CI: 2.5–97.5%**	***p*-value**	**Odds ratio**	**CI: 2.5–97.5%**	***p*-value**
Year of surgery	0.79	0.69–0.89	<0.001	1.11	1.01–1.23	<0.05	1.10	0.96–1.26	0.2
Age^*^	0.96	0.93–0.99	<0.05	1.00	0.98–1.03	0.8	1.04	1.01–1.08	<0.05
BMI	0.98	0.93–1.04	0.6	1.01	0.96–1.05	0.9	1.01	0.96–1.07	0.6
PSA	0.99	0.96–1.00	0.4	1.00	0.99–1.01	0.6	1.00	1.00–1.01	0.2
CCI 0 (reference)	1			1			1		
CCI 1	0.99	0.56–1.73	1.0	0.75	0.48–1.16	0.2	1.57	0.93–2.62	0.1
CCI 2	0.70	0.31–1.46	0.4	1.21	0.68–2.19	0.5	1.06	0.50–2.12	0.9
CCI >2	0.45	0.10–1.36	0.2	2.10	0.90–5.48	0.1	0.66	0.19–1.82	0.5

* *Continously coded*.

*CI, confidence interval; BMI, Body mass Index; PSA, Prostate-specific antigen; CCI, Charlson Comorbidity Index*.

The proportion of patients undergoing RP following a GS7 at initial biopsy increased from 54.9 to 62.4%. However, this trend did not reach statistical significance (EAPC: 6.4%, 95% CI: −3.7 to 17.5%, *p* = 0.2) (Figure 2B). After adjusting for age at surgery, BMI, CCI, and PSA, year of surgery represented an independent predictor to undergo surgery with a GS7 histology (OR: 1.11, 95% CI: 1.01–1.23, *p* < 0.05) in multivariable logistic models (Table 3).

Annual rates of patients that underwent RP following a GS8–10 at initial biopsy increased from 15.2% in 2014 to 23.7% in 2019. However, this trend was not significant (EAPC: 11.6%, 95% CI: −1.4 to 27.2%, *p* = 0.1) (Figure 2C). In multivariable logistic models (Table 3), year of surgery did not predict to undergo surgery with GS8–10 (OR: 1.10, 95% CI: 0.96–1.26, *p* = 0.2). However, older patients were more likely to undergo surgery with GS8–10 in a significant fashion (OR: 1.04, 95% CI: 1.00–1.08, *p* < 0.05).

Figure 2D presents the distribution of Gleason score groups as a percentage of the total of number of RP performed each year.

## Discussion

Taken together, our results indicate that the trend toward an inverse stage migration represents an ongoing phenomenon in our contemporary RP cohort, deriving from a European tertiary care PCa center.

First, we observed a significant decrease from 17 to 9% (EAPC: −16.4%) of the annual rate performed RP in low-risk patients (*p* < 0.05). We could illustrate that older patients (OR: 0.93) with more contemporary year of surgery (OR: 0.85) were less likely to undergo RP for low-risk PCa. This trend was not only seen in D'Amico low-risk classification but was also visible in the GS6 subgroup. The number of patients undergoing RP with GS6 histology at time of biopsy decreased from 2014 to 2019 in a significant fashion from 30 to 14.6% (EAPC: −17.6%, *p* < 0.01). Moreover, more recent year of surgery (OR: 0.79) and older age (OR: 0.96) represented protective independent predictors to undergo surgery with a GS6 histology, in multivariable models. In contrast to this finding, rising age was a predictor to undergo RP for D'Amico high-risk patients (OR: 1.02) and GS8–10 patients (OR: 1.04). This means that older patients were more likely to undergo surgery when harboring high-risk PCa, while older patients with low-risk PCa were less likely to undergo RP.

Second, annual trend analysis for intermediate-risk PCa showed a noticeable, yet not significant increase in patients undergoing RP from 51.0 to 61.5% between 2014 and 2019 (EAPC: 8.9%, *p* = 0.1). More contemporary year of surgery was an independent predictor to undergo RP in multivariable logistic models for intermediate-risk and GS7 PCa. Bearing in mind that the percentage of patients with high-risk PCa undergoing RP remained constant at 30% (EAPC: −1.7%, *p* = 0.7), an overall trend from low-risk patients to intermediate- and, in the most recent years, high-risk PCa patients undergoing RP can be seen.

These findings confirm the trend of inverse stage migration reported by other groups. For example, Budaeus et al. could demonstrate a decreasing percentage of low-risk patients undergoing RP. In this retrospective trend analysis including 8,916 patients undergoing RP between 2000 and 2009, the percentage of low-risk patients undergoing RP decreased from 66 to 35% between 2004 and 2009. In line with our findings, this trend was also visible in the subgroup of patients harboring GS6 (10). Recently, van den Bergh et al. could confirm this trend in a large-scale multicenter European retrospective analysis. With a total of 24,790 patients undergoing RP between 2000 and 2015, van den Bergh et al. showed that, in the most recent years, patients with low-risk PCa are less likely to undergo RP. In contrast, the proportion of patients undergoing RP for high-risk PCa constantly increased in this study (15), which is consistent with our findings.

Potential reasons for these observed trends could be attributed to the rising popularity of tailored, risk-adapted treatment strategies for PCa. Over the last two decades, active surveillance has been established as a standard, guideline conformance treatment option for low-risk PCa (7). With the growing adaption of active surveillance, a reduced number of patients eligible for active surveillance may have underwent RP at our institution. Loeb et al. (5) highlighted that the potential risk of overtreatment in active surveillance candidates varies from 5% to an alarming 48%, depending on the differences in the definition of histologically insignificant PCa, the investigated patient population, and biopsy practice. The application of current guidelines (7, 8) emphasizes active surveillance in low-risk PCa patients as the preferred treatment option in order to tackle overtreatment. In this respect, our data are reassuring as they indicate a more careful patient selection for surgical therapy of low-risk/GS6 PCa patients in recent years.

Besides active surveillance, focal therapy for localized PCa must be taken into consideration as a further, less-experienced modality to treat localized PCa. However, Ahdoot et al. reported that focal therapy is an encouraging modality to overcome overtreatment of localized PCa, yet robust comparative effectiveness studies and long-term oncologic outcome are warranted (16).

However, challenges of tailored, risk-adapted PCa therapy encompass not only avoidance of undertreatment but also reducing the risk of undertreating of patients with aggressive PCa requiring intensive treatment. Cooperberg et al. demonstrated that a substantial proportion of high-risk PCa is exposed to a risk of undertreatment (17). Indeed, our data show a trend toward more patients with aggressive PCa undergoing RP (biopsy GS8–10).

The absence of significance of an inverse stage migration trend in high-risk PCa patients could be explained by the limited cohort size. Furthermore, one could speculate that, in the future, with the growing number of high-risk PCa patients, this trend will be accelerated and consolidated too (18).

Our analysis has several limitations. First, the retrospective nature of our study represents an inherent limitation. However, this is common with all similar observational studies. Second, our analysis is limited by its relatively small sample size of 528 patients. Moreover, since the study is designed as a single-center analysis of a surgical high-volume center, we cannot exclude some residual bias that may have affected our results.

## Conclusion

Our study confirms that inverse stage migration represents an ongoing phenomenon in a contemporary RP cohort in a European tertiary care PCa center.

Our results demonstrate a significant decrease in the proportion of low-risk and GS6 PCa undergoing RP and a trend toward a higher proportion of intermediate-risk/GS8–10 PCa patients undergoing RP. This indicates a more precise patient selection when it comes to selecting suitable candidates for definite surgical treatment with RP.

## Data Availability Statement

The original contributions presented in the study are included in the article/supplementary material, further inquiries can be directed to the corresponding author/s.

## Ethics Statement

The studies involving human participants were reviewed and approved by Ethics Committee of University Hospital Frankfurt. The patients/participants provided their written informed consent to participate in this study.

## Author Contributions

BH and FP: manuscript writing/editing, protocol/project development, and data analysis. PM: manuscript writing/editing. MWel: data analysis. CH: data analysis. MWen: data collection. MM: data collection/data analysis. JK, PW, LK, and FC: protocol/project development. FR: manuscript editing/ project development. AB: protocol/project development and manuscript writing/editing. All authors contributed to the article and approved the submitted version.

## Conflict of Interest

The authors declare that the research was conducted in the absence of any commercial or financial relationships that could be construed as a potential conflict of interest.
